# Improving Kidney Outcomes in Patients With Nondiabetic Chronic Kidney Disease Through an Artificial Intelligence–Based Health Coaching Mobile App: Retrospective Cohort Study

**DOI:** 10.2196/45531

**Published:** 2023-06-01

**Authors:** Wei Liu, Xiaojuan Yu, Jiangyuan Wang, Tianmeng Zhou, Ting Yu, Xuyong Chen, Shasha Xie, Fuman Han, Zi Wang

**Affiliations:** 1 Department of Nephropathy Anqing Municipal Hospital Anqing China; 2 Anqing Medical Center of Anhui Medical University Anqing China; 3 Renal Division Department of Medicine Peking University First Hospital Beijing China; 4 Institute of Nephrology Peking University Beijing China; 5 Key Laboratory of Renal Disease Ministry of Health Beijing China; 6 Key Laboratory of Chronic Kidney Disease Prevention and Treatment Ministry of Education Beijing China; 7 Beijing Kidney Health Technology Co., Ltd Beijing China; 8 Fifth Clinical Medical College Anhui Medical University Hefei China

**Keywords:** chronic kidney disease, self-management, mobile apps, end-stage kidney disease, eHealth intervention, kidney, efficacy, eHealth care, dialysis, deep-learning, artificial intelligence, patient care

## Abstract

**Background:**

Chronic kidney disease (CKD) is a global health burden. However, the efficacy of different modes of eHealth care in facilitating self-management for patients with CKD is unclear.

**Objective:**

The aim of this study was to evaluate the effectiveness of a mobile app–based intelligent care system in improving the kidney outcomes of patients with CKD.

**Methods:**

Our study was a retrospective analysis based on the KidneyOnline intelligent system developed in China. Patients with CKD but not dependent on dialysis who registered on the KidneyOnline app between January 2017 and January 2021 were screened. Patients in the the KidneyOnline intelligent system group and those in the conventional care group were 1:1 matched according to their baseline characteristics. The intervention group received center-based follow-up combined with the KidneyOnline intelligent patient care system, which was a nurse-led, patient-oriented collaborative management system. Health-related data uploaded by the patients were integrated using deep learning optical character recognition (OCR). Artificial intelligence (AI)–generated personalized recipes, lifestyle intervention suggestions, early warnings, real-time questions and answers, and personalized follow-up plans were also provided. Patients in the conventional group could get professional suggestions from the nephrologists through regular clinical visits, but they did not have access to the service provided by AI and the health coach team. Patients were followed for at least 3 months after recruitment or until death or start of renal replacement therapy.

**Results:**

A total of 2060 eligible patients who registered on the KidneyOnline app from 2017 to 2021 were enrolled for the analysis. Of those, 902 (43.8%) patients were assessed for survival analysis after propensity score matching, with 451(50%) patients in the KidneyOnline intelligent patient care system group and 451(50%) patients in the conventional care group. After a mean follow-up period of 15.8 (SD 9.5) months, the primary composite kidney outcome occurred in 28 (6%) participants in the KidneyOnline intelligent patient care system group and 32 (7%) in the conventional care group, with a hazard ratio of 0.391 (95% CI 0.231-0.660; *P*<.001). Subgroup survival analysis demonstrated that the KidneyOnline care system significantly reduced the risk of composite kidney outcome, irrespective of age, sex, baseline estimated glomerular filtration rate (eGFR), and proteinuria. In addition, the mean arterial pressure (MAP) significantly decreased from 88.9 (SD 10.5) mmHg at baseline to 85.6 (SD 7.9) mmHg at 6 months (*P*<.001) in the KidneyOnline intelligent patient care system group and from 89.3 (SD 11.1) mmHg to 87.5 (SD 8.2) mmHg (*P*=.002) in the conventional CKD care group.

**Conclusions:**

The utilization of the KidneyOnline intelligent care system was associated with reduced risk of unfavorable kidney outcomes in nondiabetic patients with CKD.

## Introduction

Chronic kidney disease (CKD) is a worldwide public health concern and is increasingly becoming a global economic burden [1]. Both end-stage kidney disease (ESKD) and a reduction of estimated glomerular filtration rate (eGFR) are associated with hospitalization, cardiovascular events, and risk of death [2]. The prevalence of CKD in China was reported to be 10.8% in 2012, while only 12.5% of Chinese adults were aware of CKD as a medical problem [3]. Patients with CKD are often accompanied by comorbidities such as hypertension, diabetes, and heart disease. Providing care for patients with CKD involves a multidisciplinary team of physicians, nurses, dieticians, and social workers. Interventions to improve the outcomes of patients with CKD include lifestyle modification, antihypertensive medication, lipid modification, and glycemic control in patients with diabetes mellitus [4]. Achieving better health outcomes for patients with CKD requires patients to be aware of CKD and to engage in treatment and management plans [5]. Telehealth apps provide new opportunities to enhance self-management, behavior change, and medication adherence in a convenient way, thus reducing health complications and improving the overall kidney survival time.

There has been growing interest from physicians and other health care providers to use novel health-related mobile apps for patients in their fields of interest. Telehealth educational apps are more flexible and adaptable to patients' preferences than paper-based or in-person health education. However, according to a systematic review, CKD-related apps accounted for only 1% of the total number of available apps in 2017 [6]. In addition, as opposed to in-person health consultations, telehealth programs usually do not offer patients the chance to ask their providers or educators questions immediately, which hampers the popularity and uptake of newly developed mobile apps.

Up to now, most studies describing the effectiveness of eHealth interventions on patients with CKD were from North America [7]. Moreover, the studies had relatively small sample sizes and only investigated changes in participants’ clinical parameters in a short period of time. Importantly, there has been a lack of studies assessing eHealth interventions using hard kidney end points. The objective of this study is to evaluate the effectiveness of a novel nurse-led, real-time communicating, app-based intelligent patient care system in China between 2017 and 2021 from real-world data.

## Methods

### Study Design and Participants

This study was a retrospective cohort analysis based on a mobile app called KidneyOnline in China. The KidneyOnline app offered a platform with free materials and resources about CKD. Patients received recruitment information about the KidneyOnline app within WeChat; subsequently, they downloaded the app and became active app users. The KidneyOnline intelligent patient care system was embedded in the app, and users made their own decision about whether to join the intelligent care system. Informed patient consent forms were signed by the patients within the KidneyOnline app. All app users between January 2017 and January 2021 were screened according to the recruitment criteria, and eligible patients were grouped according to their original choice.

Patients were enrolled if they (1) were over 18 years old; (2) fulfilled the diagnosis of CKD (ie, eGFR <60 mL/min/1.73 m^2^ or eGFR < 90 mL/min/1.73 m^2^ but with albuminuria or hematuria for at least 3 months or as defined using other clinically indicated criteria); (3) uploaded 2 pieces of data upon registration with an interval of at least 3 months; (4) were free of dialysis, with a baseline eGFR >15mL/min/1.73 m^2^; and (5) were able and willing to provide informed consent. Patients were excluded if (1) they or their relatives were unable to use a smartphone, (2) they intended to start dialysis or have a kidney transplant within the next 3 months, (3) there was a lack of baseline or follow-up data, or (4) they refused to communicate with the health coach team. Study participants were followed for at least 3 months after recruitment until death or the start of renal replacement therapy.

### Intervention

**Figure 1:**
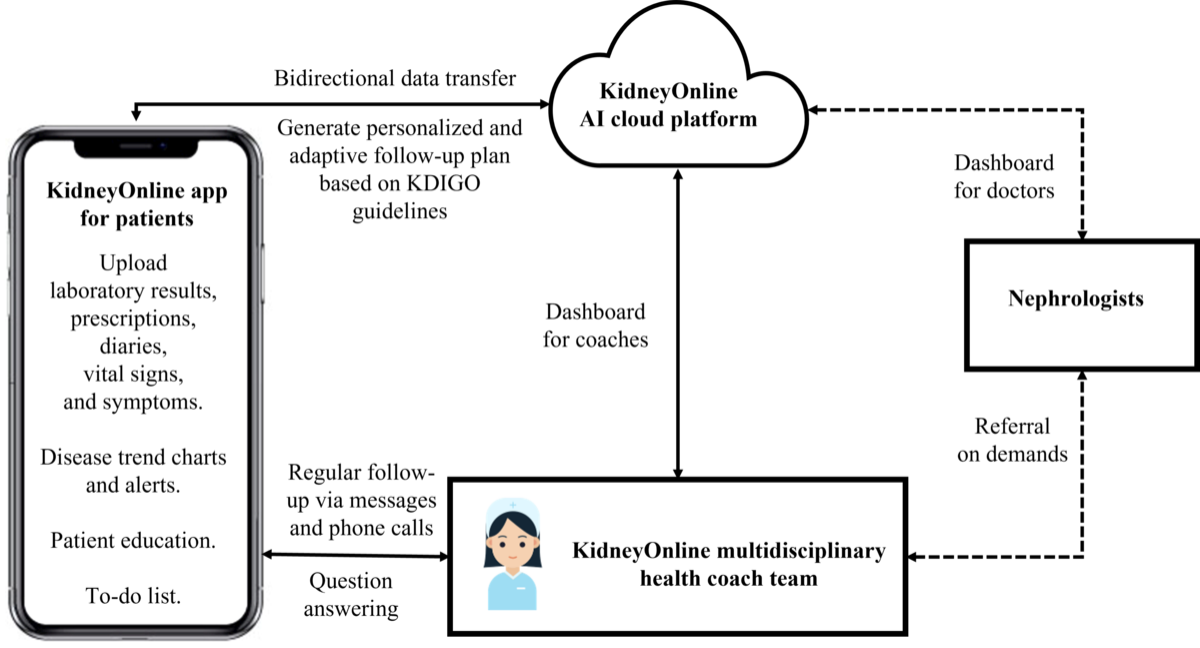
Framework of KidneyOnline intelligent care system. AI: artificial intelligence; KDIGO: Kidney Disease: Improving Global Outcomes (clinical practice guidelines).

The KidneyOnline intelligent patient care system was a nurse-led, patient-oriented collaborative management system as an adjunct to regular clinic visits for patients with CKD (Figure 1). The intelligent system was empowered by artificial intelligence (AI) and a health coach team, which consisted of a group of experienced nurses trained by nephrologists, dieticians, and social workers. The system consisted of a smartphone app for patients, a web-based clinical dashboard app for health care providers, and a data server for information management (Figure 1).

**Figure 2:**
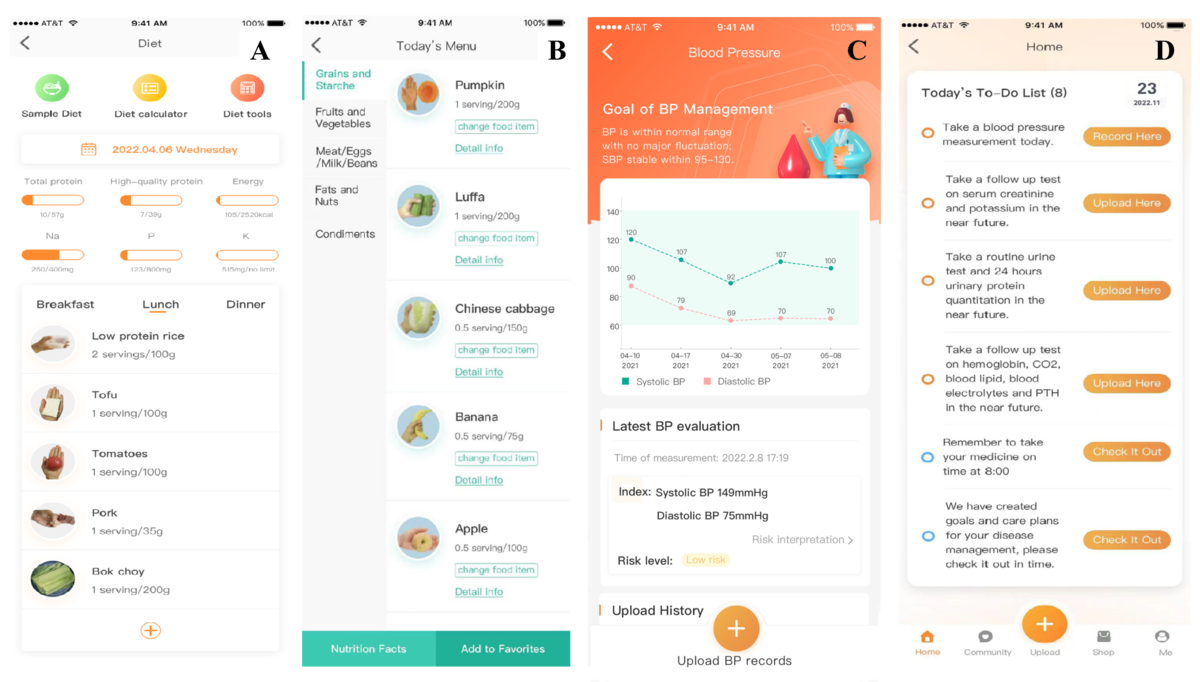
Artificial intelligence (AI)–generated personalized recipes, blood pressure (BP) monitoring. and personal checklist dashboards of the KidneyOnline app.

Participants in the KidneyOnline intelligent patient care group were able to experience at least 5 aspects of service provided by the system, which are detailed in Textbox 1 (Figure 2).

Patients in the conventional care group only had access to the health-related educational materials provided by the app. They could get professional suggestions from the nephrologists through regular clinical visits, but they did not have access to the service provided by AI and the health coach team.

**The 5 aspects of service provided to patients by the KidneyOnline system.**

1. Interpretation of disease condition and corresponding guidance. The intelligent care system helped explain patients’ diagnoses and medication prescriptions, analyze and interpret lab results, and remind patients about medication precautions. The system also provided guidance on lifestyle interventions, including diet, exercise, and sleep. Patients could receive artificial intelligence (AI)–generated personalized recipes based on their disease condition and food preferences, and they were able to make food diaries to see if the nutrition requirements were met.
2. Regular check-ups. The intelligent care system followed each patient regularly to check if the patient was in good condition, assure if the recent BP was well-controlled, evaluate whether the patient’s current medication was reasonable, and assess whether the patient’s dietary intake was reasonable.
3. Early warnings. Through the algorithm, the intelligent system was able to identify risks associated with lab results and certain medications and make early warnings to minimize these risks. If abnormal lab results requiring immediate attention were identified, the system would send a medical alert to both the patient and the health coach. The health coach would take the initiative to contact the patient and make suggestions, including reminding the patient about medications, providing guidance on diet, exercise, and other lifestyle aspects, and reminding the patient to consult with doctors.
4. Real-time question-and-answer fields empowered by knowledge graphs. We established a renal knowledge graph according to the Kidney Disease: Improving Global Outcomes (KDIGO) guidelines for chronic kidney disease (CKD), which provided intelligent search and human-computer dialogues to enhance the efficiency and professional competency of the health coach team. Empowered by the graphs, the health coaches promptly provided real-time answers to any questions the patients had (Multimedia Appendix 1). These include laboratory results analysis, medication precautions, dietary guidance, exercise guidance, advice on coping with unexpected situations, advice on the correct way to take medicine to avoid kidney injuries, and so forth.
5. Clinical reminders. According to the patient's overall condition, the intelligent system could generate a personalized follow-up plan, reminding the patient to go to the doctor at regular intervals, helping them sort out test items they need to finish, and checking follow-up results to confirm whether the follow-up visit was completed.

### Data Collection

The foundation of this intelligent system was built upon the structurization of patients’ health-related data using deep learning optical character recognition (OCR). Conventionally, patients’ health data came from a variety of sources, including (1) patients’ self-reported signs and symptoms; (2) data from intelligent home devices such as sphygmomanometer, and (3) patients’ past medical history, clinical notes, drug prescriptions, lab results, pathological reports, imagological exams, and so on. In the KidneyOnline intelligent system, patients uploaded those data simply by taking photos, and the intelligent system extracted the data efficiently by utilizing deep learning OCR. Combined with manual verification, a structured database was built so that patients’ health-related data could be integrated and analyzed quantitatively.

The participants took photos of their medical records, laboratory test results, and clinical prescriptions and uploaded them on the mobile app. All electronic data and photographs were uploaded instantly to a secure, cloud-based server. To comply with security and privacy regulations, patients’ smartphones were password-protected, and data were encrypted. Only the researchers were able to access the data on a cloud storage platform.

### Outcomes

The primary outcome of our study was the first occurrence of either a 30% decrease in eGFR or an incidence of ESKD. Secondary end points included changes in 24-hour proteinuria and changes in mean arterial pressure (MAP). eGFR results were calculated by using the Chronic Kidney Disease Epidemiology Collaboration (CKD-EPI) formula. Blood pressure data were collected through the app based on home blood pressure measurements uploaded by the patients, using either mercury or an electronic sphygmomanometer.

### Ethics Approval

This study was approved by the local research ethics board from Anqing Municipal Hospital (2022-033) and was conducted according to the Declaration of Helsinki.

### Statistical Analysis

#### Reporting Descriptive Data

The distributional properties of data were expressed as mean (SD) for continuous variables with a normal distribution or median (IQR) for variables with a skewed distribution. For continuous data, the independent or paired Student *t* test was used for within-group and between-group comparisons; for categorical variables with percentages, the chi-square or McNemar test was used. Clinical parameters including 24-hour proteinuria and MAP during the follow-up were compared using Student *t* tests (for normally distributed continuous variables) and Wilcoxon signed-rank tests (for nonnormally distributed continuous variables).

#### Propensity Score Matching

To balance the confounding factors between the KidneyOnline intelligent patient care system group and the conventional CKD care group, 1:1 propensity score matching (PSM) was performed using nearest neighbor algorithms with a 0.9 caliper width of 0.02 pooled standard deviations. Matching was based on baseline characteristics of age, sex, BMI, baseline eGFR, MAP, and proteinuria levels.

#### Assessment of the Benefits of KidneyOnline

For survival analysis, both the Kaplan-Meier method and Cox proportional hazards model were used. The Kaplan-Meier method was applied to evaluate the cumulative incidence of primary outcomes in both groups following PSM. Additionally, the Cox proportional hazards model was utilized to identify predictive factors associated with the outcomes. For matched data after PSM, the Cox proportional hazards model with gamma frailty was used. All missing information was treated as missing data without imputation. Subgroup analyses were performed after stratifying according to the median age (<33 years vs ≥33 years), sex, kidney function (eGFR <60 mL/min/1.73 m^2^ vs ≥60 mL/min/1.73 m^2^), and proteinuria (<1 g/24 h vs ≥1g/24 h, <3 g/24 h vs ≥3 g/24 h), as well as the median value of baseline MAP (<88.7 mmHg vs ≥88.7 mmHg). Statistical analyses were performed using Python and Lifelines, an open-access survival analysis library written in Python. A 2-sided *P*<.05 was considered statistically significant.

## Results

### Baseline Characteristics

**Figure 3:**
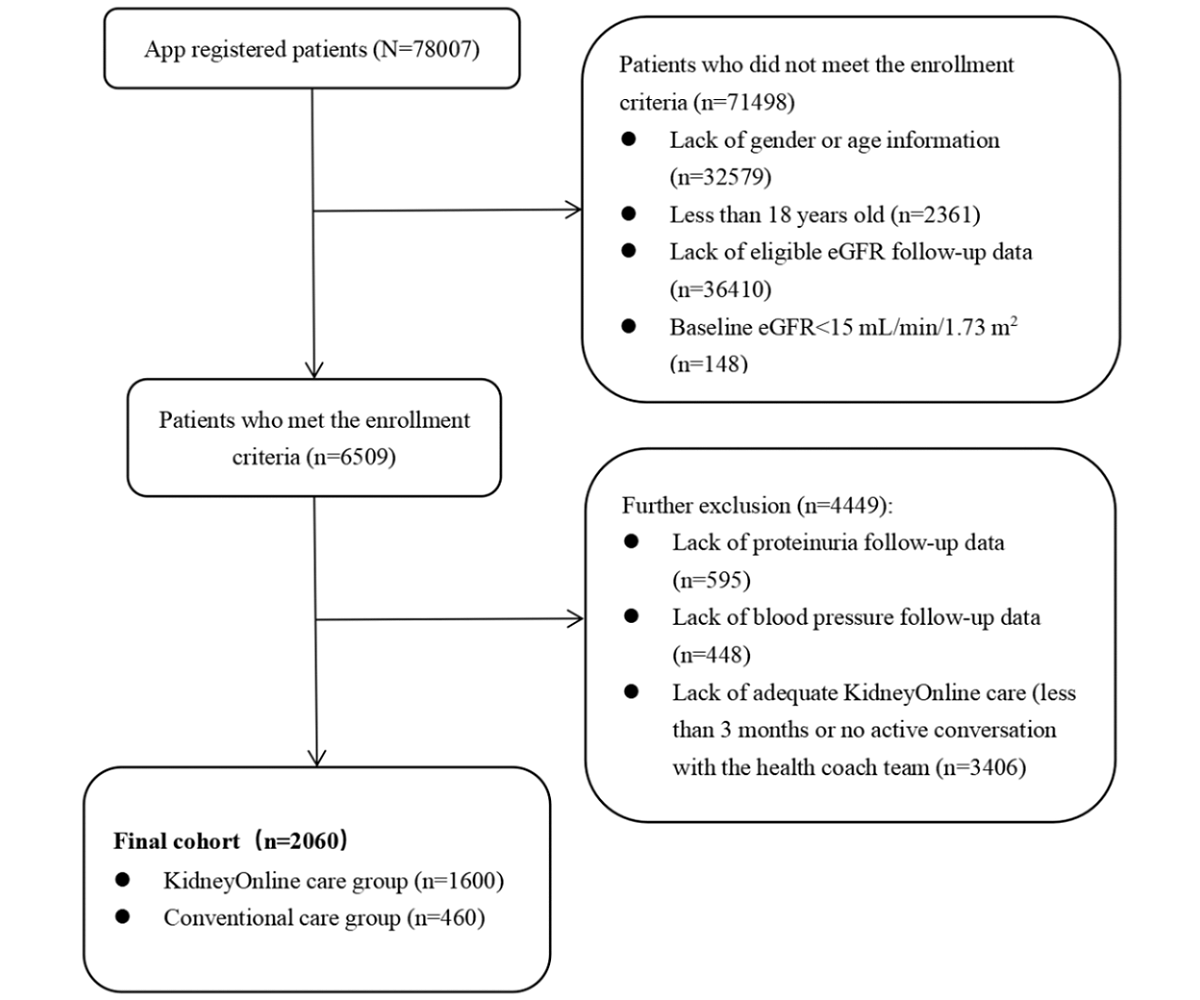
Flow diagram of patient enrollment for the analysis. eGFR: estimated glomerular filtration rate.

Between January 2017 and January 2021, 78,007 potentially eligible patients were screened. Among them, 2060 (2.6%) were eligible for our analysis according to the inclusion and exclusion criteria. There were 1600 (77.7%) patients in the KidneyOnline intelligent patient care system group and 460 (22.3%) patients in the conventional care group (Figure 3). Among the 2060 patients enrolled in our study, the mean age was 35.6 (SD 9.5) years, 1175 (57%) were female, and they had an average BMI of 22.7 (SD 4.2) kg/m^2^ (Table 1). Upon registration, the mean eGFR was 88.6 (SD 31.3) mL/min/1.73 m^2^, and the mean proteinuria level was 1.4 (SD 1.8) g/24 h. The average MAP was 89 (SD 11) mmHg. During the follow-up period, 1539 (75%) patients were treated with a renin-angiotensin-aldosterone system blocker (RASB), 524 (25%) were treated with steroids, and 418 (20%) were treated with immunosuppressants. Compared with patients in the conventional care group, patients in the KidneyOnline intelligent patient care system group had a more favorable BMI (mean 22.5, SD 4 kg/m^2^ vs mean 23.2, SD 4.8 kg/m^2^; *P*=.004) and lighter 24-hour proteinuria (median 0.7, IQR 0.3-1.6 g/24 h vs median 0.9, IQR 0.3-1.9 g/24 h; *P*=.033) but worse baseline kidney function (mean 87.8, SD 31.3 mL/min/1.73 m^2^ vs mean 91.5, SD 31.4 mL/min/1.73 m^2^; *P*=.024). There were no significant differences in the context of RASB and steroids or immunosuppressants usage between the 2 groups (Table 1).

**Table 1:** Baseline and follow-up characteristics of patients in the whole cohort.

Demographics			Total (N=2060)		Conventional care group (n=460)		KidneyOnline care group (n=1600)		*P* value
Age (years), mean (SD)			35.6 (9.5)		35.3 (9.6)		35.8 (9.5)		.32
**Sex, n (%)**									
	Female	1175 (57)		248 (53.9)		927 (57.9)		.12	
	Male	885 (43)		212 (46.1)		673 (42.1)		.12	
BMI (kg/m^2^), mean (SD)			22.7 (4.2)		23.2 (4.8)		22.5 (4)		.004
**Etiology of CKD^a^, n (%)**									
	IgA^b^ nephropathy or IgA vasculitis	945 (45.9)		182 (39.6)		763 (47.7)		.002	
	Membranous nephropathy	185 (9)		49 (10.7)		136 (8.5)		.18	
	Focal segmental glomerular sclerosis	63 (3.1)		13 (2.8)		50 (3.1)		.86	
	Hypertensive nephropathy	53 (2.6)		23 (5)		30 (1.9)		<.001	
	Diabetic nephropathy	7 (0.3)		1 (0.2)		6 (0.4)		.95	
	Other types of nephritis	246 (11.9)		50 (10.9)		196 (12.2)		.47	
	Unknown etiology	559(27.1)		141(30.7)		418(26.1)		.062	
	Kidney transplant recipient	2 (0.1)		1 (0.2)		1 (0.1)		.93	
**Laboratory results**									
	Baseline eGFR^c^ (mL/min/1.73 m^2^), mean (SD)	88.6 (31.3)		91.5 (31.4)		87.8 (31.3)		.024	
	CKD stage 3-4, n (%)	443 (21.5)		88 (19.1)		355 (22.2)		.18	
	Baseline proteinuria (g/24 h), median (IQR)	0.8 (0.3-1.7)		0.9 (0.3-1.9)		0.7 (0.3-1.6)		.033	
	Baseline MAP^d^ (mmHg), mean (SD)	88.6 (10.9)		89.5 (11.1)		88.4 (10.8)		.051	
**Drug therapies, n (%)**									
	RASB^e^	1539 (74.7)		333 (72.4)		1206 (75.4)		.19	
	Steroids	524 (25.4)		127 (27.6)		397 (24.8)		.23	
	Immunosuppressants	418 (20.3)		94 (20.4)		324 (20.3)		.93	

^a^CKD: chronic kidney disease.

^b^IgA: immunoglobulin A.

^c^eGFR: estimated glomerular filtration rate.

^d^MAP: mean arterial pressure.

^e^RASB: renin-angiotensin-aldosterone system blocker.

### Benefits of KidneyOnline Care System for the Whole Population

After a mean follow-up of 18.1 (SD 9.5) months, the primary composite kidney outcome occurred in 121 (8%) participants in the KidneyOnline intelligent patient care system group and 33 (7%) in the conventional care group, with 86 (5%) versus 21 (5%) and 35 (2%) versus 12 (3%) participants reaching a 30% eGFR decrease and ESKD, respectively.

Multivariate Cox regression with stepwise procedures was used to analyze the risk factors of composite kidney outcome. After adjustments for age, gender, baseline eGFR, proteinuria, MAP, and use of RASB, steroids, and immunosuppressants, individuals in the KidneyOnline care group were less likely to progress to ESKD compared with the conventional group, with a hazard ratio of 0.375 (95% CI 0.221-0.638; *P*<.001) (Multimedia Appendix 2).

### Characteristics of the Individuals After PSM

A 1:1 PSM analysis was performed to balance the selection bias between the 2 groups. A total of 902 patients with 451 (50%) patients in each group were successfully matched. In total, the average age was 35.5 (SD 9.4) years, and 54% (n=487) were female. The average BMI was 22.9 (SD 3.8) kg/m^2^. The baseline MAP was 89.1 (SD 10.8) mmHg, and the 24-hour proteinuria was 0.8 (IQR 0.3-1.8) g/24 h. The baseline eGFR of the 902 patients was 91.9 (SD 30.7) mL/min/1.73 m^2^ (Table 2). Overall, there were 672 (75%) patients who received RASB, 232 (26%) who received steroids, and 197 (22%) who received immunosuppressant therapy. There were no significant differences in laboratory results and treatments between the KidneyOnline intelligent patient care system and the conventional care group at baseline (Table 2).

**Table 2:** Baseline and follow-up characteristics of patients after propensity score matching (PSM).

Demographics		Total (N=902)	Conventional care group (n=451)	KidneyOnline care group (n=451)	*P* value
Age (years), mean (SD)		35.5 (9.4)	35.27 (9.6)	35.67 (9.2)	.51
**Sex, n (%)**					
	Female	487 (54)	244 (54.1)	243 (53.9)	>.99
	Male	415 (46)	207 (45.9)	208 (46.1)	>.99
BMI (kg/m^2^), mean (SD)		22.9 (3.8)	22.9 (4.0)	23.0 (3.6)	.90
**Etiology of CKD^a^, n (%)**					
	IgA^b^ nephropathy or IgA vasculitis	386 (42.8)	181 (40.1)	205 (45.5)	.13
	Membranous nephropathy	95 (10.5)	45 (10)	50 (11.1)	.64
	Focal segmental glomerular sclerosis	23 (2.5)	12 (2.7)	11 (2.4)	>.99
	Hypertensive nephropathy	29 (3.2)	22 (4.9)	7 (1.6)	.006
	Diabetic nephropathy	4 (0.4)	1 (0.2)	3 (0.7)	.63
	Other types of nephritis	99 (11)	48 (10.6)	51 (11.3)	.84
	Unknown etiology	265 (29.4)	141 (31.3)	124 (27.5)	.24
	Kidney transplant recipient	1 (0.1)	1 (0.2)	0 (0)	>.99
**Laboratory results**					
	Baseline eGFR^c^ (mL/min/1.73 m^2^), mean (SD)	91.9 (30.7)	91.5 (31.3)	92.3 (30.2)	.63
	CKD stage 3-4, n (%)	163 (18.1)	86 (19.1)	77 (17.1)	.49
	Baseline proteinuria (g/24 h), median (IQR)	0.8 (0.3-1.8)	0.8 (0.3-1.8)	0.8 (0.3-1.6)	.29
	Baseline MAP^d^ (mmHg), mean (SD)	89.1 (10.8)	89.3 (11.0)	88.9 (10.5)	.56
**Drug therapies**					
	RASB^e^	672 (74.5)	327 (72.5)	345 (76.5)	.20
	Steroids	232 (25.7)	123 (27.3)	109 (24.2)	.32
	Immunosuppressants	197 (21.8)	93 (20.6)	104 (23.1)	.41

^a^CKD: chronic kidney disease.

^b^IgA: immunoglobulin A.

^c^eGFR: estimated glomerular filtration rate.

^d^MAP: mean arterial pressure.

^e^RASB: renin-angiotensin-aldosterone system blocker.

### Benefits of the KidneyOnline Care System for Propensity-Matched Individuals

**Figure 4:**
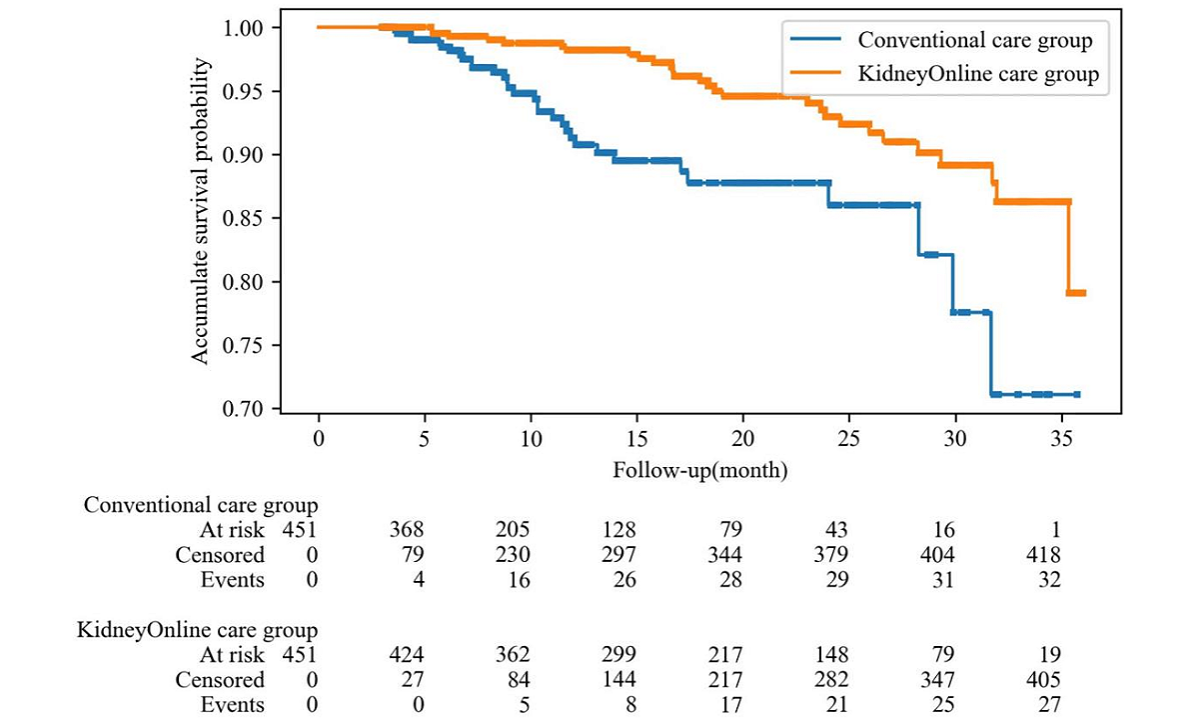
Kaplan-Meier curve of primary composite kidney outcomes.

After a mean follow-up period of 15.8 (SD 9.5) months, the primary composite kidney outcome occurred in 28 (6%) participants in the KidneyOnline intelligent patient care system group and in 32 (7%) in the conventional care group, with a hazard ratio (HR) of 0.391 (95% CI 0.231-0.661; *P*<.001) (Figure 4), with 20 (4%) versus 20 (4%) and 8 (2%) versus 12 (3%) participants reaching a 30% decrease in eGFR and ESKD, respectively. A subgroup analysis showed that the KidneyOnline intelligent patient care system significantly reduced the risk of composite kidney outcome in all subgroups stratified by age (<33 vs ≥33 years), sex, kidney function (eGFR <60 vs ≥60 mL/min/1.73 m^2^), and proteinuria (<1 vs ≥ g/24 h, <3 vs ≥3 g/24 h). However, the KidneyOnline intelligent patient care system improved the composite kidney outcomes in patients with elevated levels of MAP, but it did not improve composite kidney outcomes in the normal MAP group (Multimedia Appendix 3).

### Changes in MAP During Follow-up

Since the KidneyOnline intelligent patient care system showed different effects in different MAP groups, we further analyzed the MAP during follow-up between the KidneyOnline group and the conventional CKD care group. The baseline MAP values were similar between the 2 groups. The MAP significantly decreased from 88.9 (SD 10.5) mmHg at baseline to 85.6 (SD 7.9) mmHg at 6 months (*P*<.001) in the KidneyOnline group and from 89.3 (SD 11.1) mmHg to 87.5 (SD 8.2) mmHg (*P*=.002) in the conventional CKD care group. After 6 months, the MAP in the KidneyOnline group participants remained at a lower level compared with that in the conventional CKD care group (Multimedia Appendix 4A). This trend was also observed when participants were stratified according to the median value of MAP (88.7 mmHg) (Multimedia Appendix 4B,C).

### Changes in Proteinuria During Follow-up

The mean level of 24-hour proteinuria in the KidneyOnline intelligent patient care system group was not significantly different from the conventional CKD care group at baseline (1.4 vs 1.5 g/24 h, *P*=.36). During the follow-up, the mean level of 24-hour proteinuria decreased to 0.8 g/24 h in the KidneyOnline intelligent patient care system group and 0.8 g/24 h in the conventional care group at end of 12 months. There was no significant difference in the mean level of 24-hour proteinuria between the 2 groups by the end of 24 months (0.7 vs 0.7 g/24 h, *P*=.92) (Multimedia Appendix 5).

## Discussion

### Principal Findings

In this study, we described a nurse-led, smartphone-based patient-oriented system designed to help disease management in patients living with stages 1 to 4 of CKD. Our findings demonstrate that this intelligent care system was associated with better blood pressure control and a reduced risk of kidney failure. This provided a novel strategy for promoting a healthy lifestyle and improving kidney prognosis in patients with CKD, regardless of their scheduled consultations.

### Comparisons to Prior Work

The incidence and prevalence of CKD have been persistently increasing because of the aging population, who experience a high incidence of metabolic disorders such as hypertension, diabetes, and obesity [8-10]. There have been efforts to find innovative and efficient ways to improve patient outcomes, but the results have been conflicting due to varied intervention facets and intensities. Few studies showed improvement in hard kidney end points or eGFR change. The intervention conducted in the Multifactorial Approach and Superior Treatment Efficacy in Renal Patients With the Aid of Nurse Practitioners (MASTERPLAN) study [11] initially reported negative results of the strict implementation of CKD guidelines to patients with CKD in decreasing the risk of ESKD. Nevertheless, after a prolonged follow-up, Peeters et al [12] demonstrated that additional support by nurse practitioners, including lifestyle intervention, use of mandatory medication, and implementation of CKD guidelines reduced the risk of composite renal endpoints (death, ESKD and 50% increase in serum creatinine) by 20%. The care management performed by nurse practitioners at least quarterly resulted in a decrease in eGFR of 0.45 mL/min/1.73 m^2^ per 1.73 m^2^ per year in the intervention group compared with the control group [12]. In a study conducted in Taiwan, Barrett et al [13] showed that multidisciplinary education according to the National Kidney Foundation's Kidney Disease Outcomes Quality Initiative (NKF/KDOQI) guidelines decreased the incidence of dialysis and reduced mortality in predialysis patients with CKD after a mean follow-up of 11.7 months. Recently, in their 3-month-prospective study, Li et al [14] reported that patients with CKD who received diet, exercise, and self-management education delivered via a wearable device and smartphone app experienced a slower rate of eGFR decline than those given only conventional care. These studies suggested that beyond traditional nephrologist-centered clinical consultations, proper education in combination with the careful management of patients with CKD could potentially improve kidney outcomes even in the short term. In the KidneyOnline intelligent patient care system, patients were educated by nurses who received training according to the Kidney Disease: Improving Global Outcomes (KDIGO) guidelines. Other interventions including lifestyle intervention and education were all integrated into the KidneyOnline care system. Our study confirmed previous findings that nurse care could improve the renal outcomes of patients with CKD via patient education, disease interpretation, and lifestyle intervention.

### Analysis of Findings

Our study demonstrated that the KidneyOnline care system reduced the risk of composite kidney end points, irrespective of age, baseline eGFR, and proteinuria. This effect was multifactorial. One of the main underlying reasons for this effect was the well-controlled blood pressure, which was significantly lower at 6 months compared with that at the time of enrollment in the KidneyOnline care group, and it remained at a lower level throughout the follow-up period. In China, the prevalence of hypertension is higher in patients with CKD compared with the general population, but awareness of hypertension in patients with CKD was reported to be 80.7% in 2018[15]. Previous studies have shown that adequate control of blood pressure was suboptimal in patients with CKD [16,17]. In fact, refractory hypertension in patients was largely attributed to inadequate adherence to prescribed medication [18]. Low medication adherence was associated with CKD progression [19,20]. It has been established that well-designed mobile apps could effectively improve medication adherence in cardiovascular disease [21,22]. In the KidneyOnline care system group, the trend graph and brief report about blood pressure, real-time online questions and answers, and medication intake suggestions were all potential factors that might have contributed to patients’ good adherence. Moreover, constant online communication and laboratory test reminders possibly prompted participants’ clinical visits to adjust their medication regimens in time, thus reducing the risk of adverse drug reactions. Finally, healthy lifestyle modifications, including sufficient physical activity, proper BMI control, smoking cessation, and healthy diet habits, were reported to help slow the progression of CKD in patients with preserved kidney function [23]. Participants joining the KidneyOnline care group often gave positive feedback on the smart reminder for helping them organize their diets and make healthy lifestyle choices.

Interestingly, there was an improvement in proteinuria in both the KidneyOnline intelligent care group and the conventional care group. This may be attributed to the KidneyOnline app's educational materials on the benefits of a low-salt diet. In a typical Chinese diet, the salt intake is often much higher than recommended. The KidneyOnline app enabled patients from both groups to access free educational materials on the importance of a low-salt diet, as well as tips on limiting salt intake. Moreover, the KidneyOnline app offered a variety of low-salt diet recipes, which might have helped increase patients’ compliance with a low-salt diet. Additionally, there was a high percentage of patients with glomerulonephritis, with 25.7% (n=232) being treated with steroids. The use of steroids and immunosuppressants is another important factor leading to the improvement in proteinuria.

The rate of smartphone ownership has exploded in China during the last 10 years. Telehealth apps provide new opportunities to enhance self-management, behavior change, and medication adherence not only for people living in urban areas but also for those in rural areas. As Duan et al [24] have shown, the prevalence of CKD in China’s rural areas was 16.4% between 2015 and 2017. Education level, personal income, alcohol consumption, and hypertension were all risk factors associated with insufficient kidney function. The KidneyOnline care system provides a web-based solution for patient-centered care and helps reduce the time and cost associated with long travels to seek medical advice. Thus, our mode of care could facilitate patients’ self-management in a cost-effective manner, especially in areas facing a shortage of medical resources.

### Strengths and Limitations

To our knowledge, this study was the first to assess the efficacy of a smartphone-based, nurse-led, patient-oriented management system for patients with CKD across China using hard kidney end points. The 4-year observational data from the real world demonstrated the efficacy of this newly developed system. Inevitably, our study had several limitations. First, there were over 70,000 user registrations on our app. Only 2060 patients’ records fulfilled our criteria and were eligible for analysis. This could have resulted in selection bias in both groups. Patients with a stronger sense of self-management and motivation were more likely to be included in this study. Second, patients who had severe symptoms or advanced pathological lesions were less likely to choose our patient care system; instead, they received treatments in large hospitals on their first visits. Moreover, patients with minor renal impairment could potentially have a lower probability of joining the KidneyOnline care system. In addition, due to the cost associated with the KidneyOnline care system, patients who joined the system probably had a higher economic status compared with patients in the control group. Nonetheless, we have shown that patients in the KidneyOnline care group experienced better prognoses in terms of composite renal outcomes, irrespective of their age, baseline eGFR, and proteinuria. More data are needed to illustrate the efficacy of our mode of care in those with more impaired kidney function, as well as those with very slight renal impairment. Third, we did not analyze other possible end points related to the utilization of the KidneyOnline care system, such as cardiovascular comorbidities and hospitalization frequencies, which would help us better evaluate our mode of management for patients with CKD. Additionally, the participants were young and normotensive; there was a high prevalence of glomerulonephritis but a limited number of diabetes cases, and these factors may have limited the feasibility of the KidneyOnline care system for patients with CKD caused by diabetic nephropathy. It is also important to note that our study was conducted in China; because the education and economic levels of patients with CKD vary across countries, a new mode of patient care based on mobile phones in other countries is anticipated.

### Conclusion

We developed a smartphone-based, nurse-led, patient-oriented management system to facilitate health care for patients with nondiabetic CKD in China. Through multifaceted patient care, our mode of patient management was associated with a reduced risk of composite kidney outcomes. This strengthened the evidence of telehealth interventions to promote kidney health and long-term management for patients with CKD.

## Appendix

Multimedia Appendix 1 Demonstration of KidneyOnline dashboard for patients and health coach team.

Multimedia Appendix 2 Multivariate Cox regression analysis of composite kidney outcome (before patient matching). eGFR: estimated glomerular filtration rate; MAP: mean arterial pressure; RASB: renin-angiotensin-aldosterone system blocker.

Multimedia Appendix 3 Forest plot of the comparison between the 2 different modes of care in different subgroups (after patient matching). eGFR: estimated glomerular filtration rate; MAP: mean arterial pressure.

Multimedia Appendix 4 Changes in mean arterial pressure (MAP) during follow-up in the whole population and subgroups.

Multimedia Appendix 5 Changes in 24-hour proteinuria in the 2 matched groups of patients after propensity score matching (PSM) during follow-up.

## Abbreviations

AI: artificial intelligence
CKD: chronic kidney disease
CKD-EPI: Chronic Kidney Disease Epidemiology Collaboration
eGFR: estimated glomerular filtration rate
ESKD: end-stage kidney disease
HR: hazard ratio
KDIGO: Kidney Disease: Improving Global Outcomes
MAP: mean arterial pressure
NKF/KDOQI: National Kidney Foundation's Kidney Disease Outcomes Quality Initiative
OCR: optical character recognition
PSM: propensity score matching
RASB: renin-angiotensin-aldosterone system blocker

## Data Availability

The data sets analyzed in this study are not publicly available due to business confidentiality reasons but are available from the corresponding author upon reasonable request.
