# Supplementary figures and images for: Improving Kidney Outcomes in Patients With Nondiabetic Chronic Kidney Disease Through an Artificial Intelligence–Based Health Coaching Mobile App: Retrospective Cohort Study
Source: JMIR Mhealth Uhealth. 2023 Jun 1;11:e45531. doi: 10.2196/45531 (PMC10273040; doi:10.2196/45531)

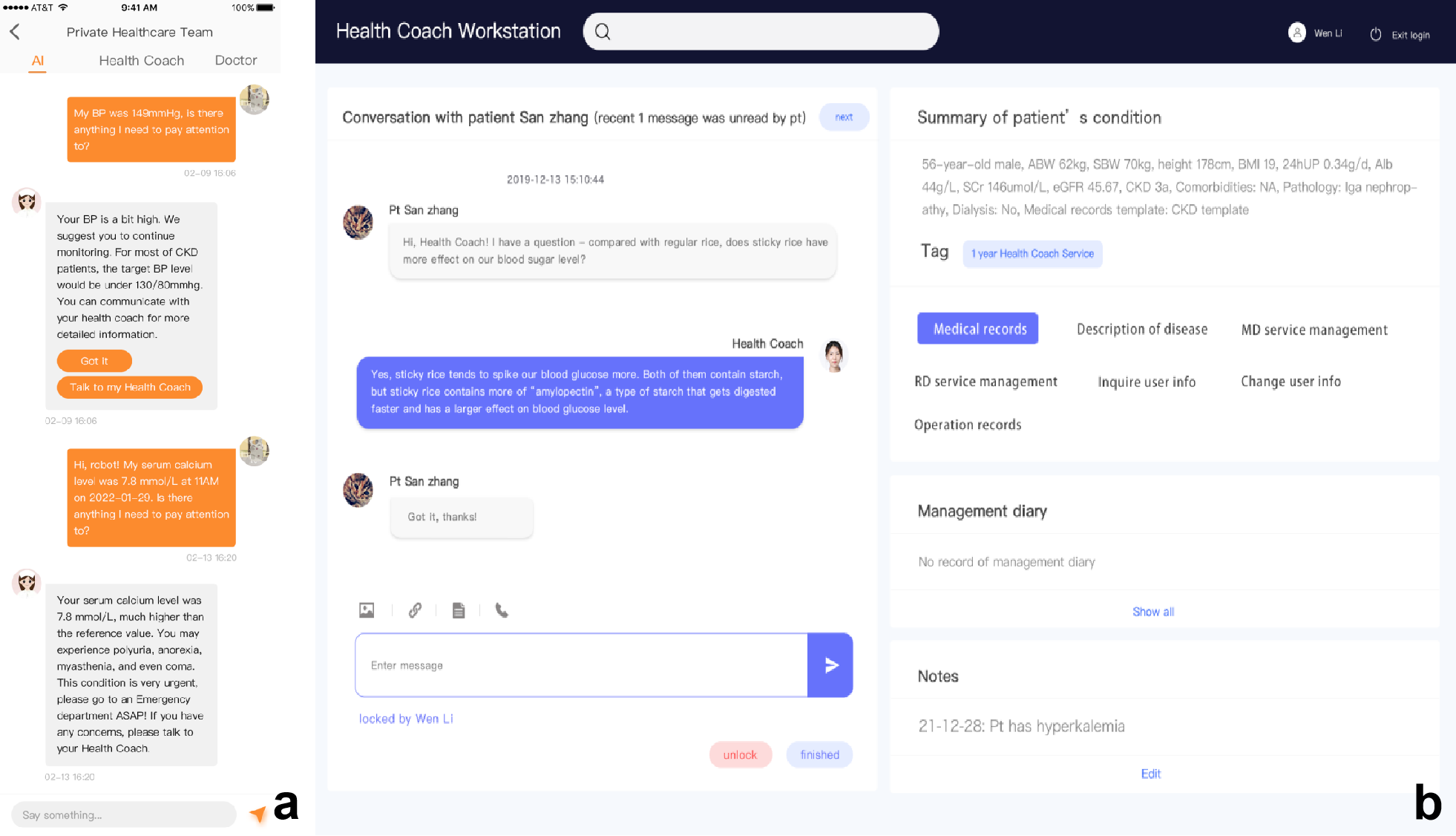

Supplement: Multimedia Appendix 1 [file mhealth_v11i1e45531_app1.png]

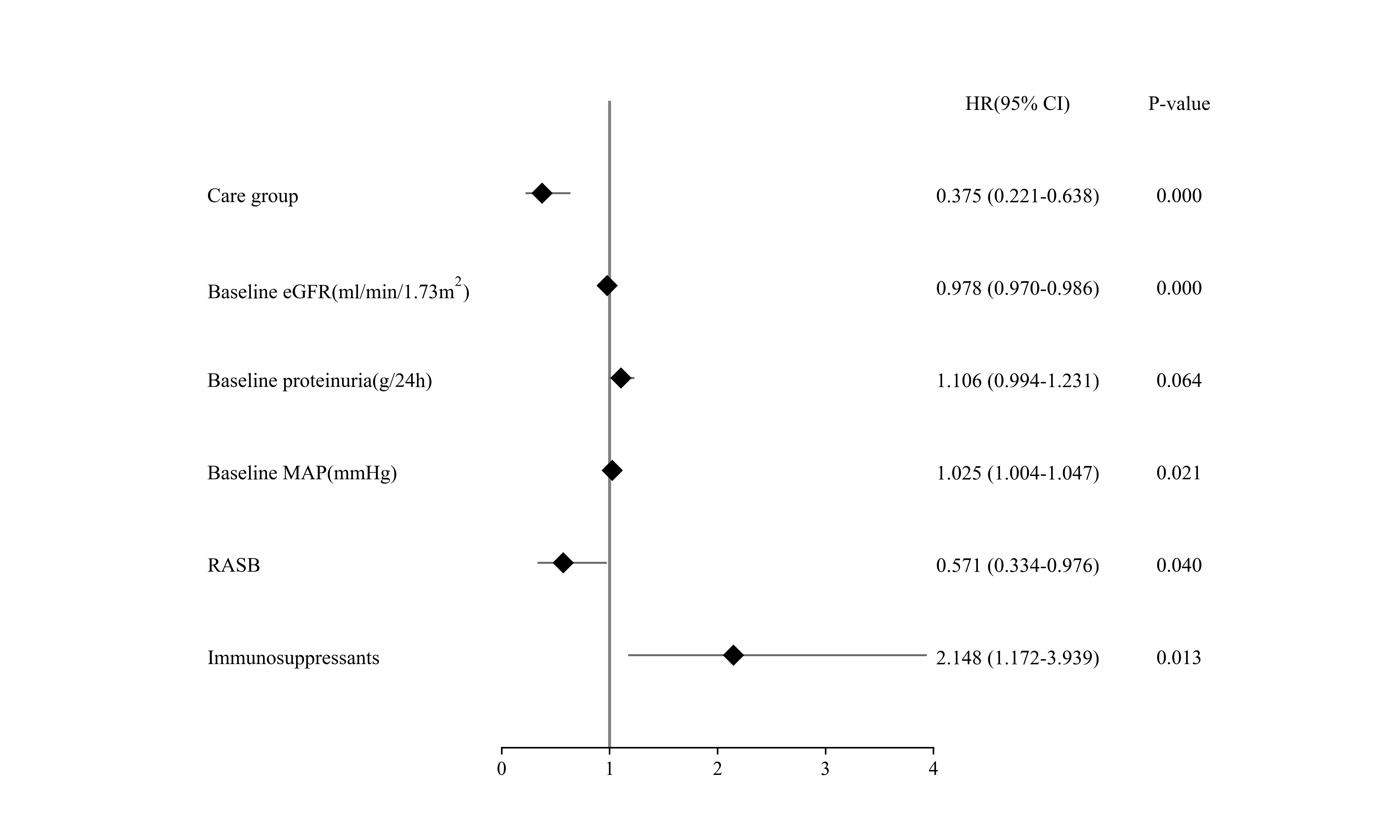

Supplement: Multimedia Appendix 2 [file mhealth_v11i1e45531_app2.png]

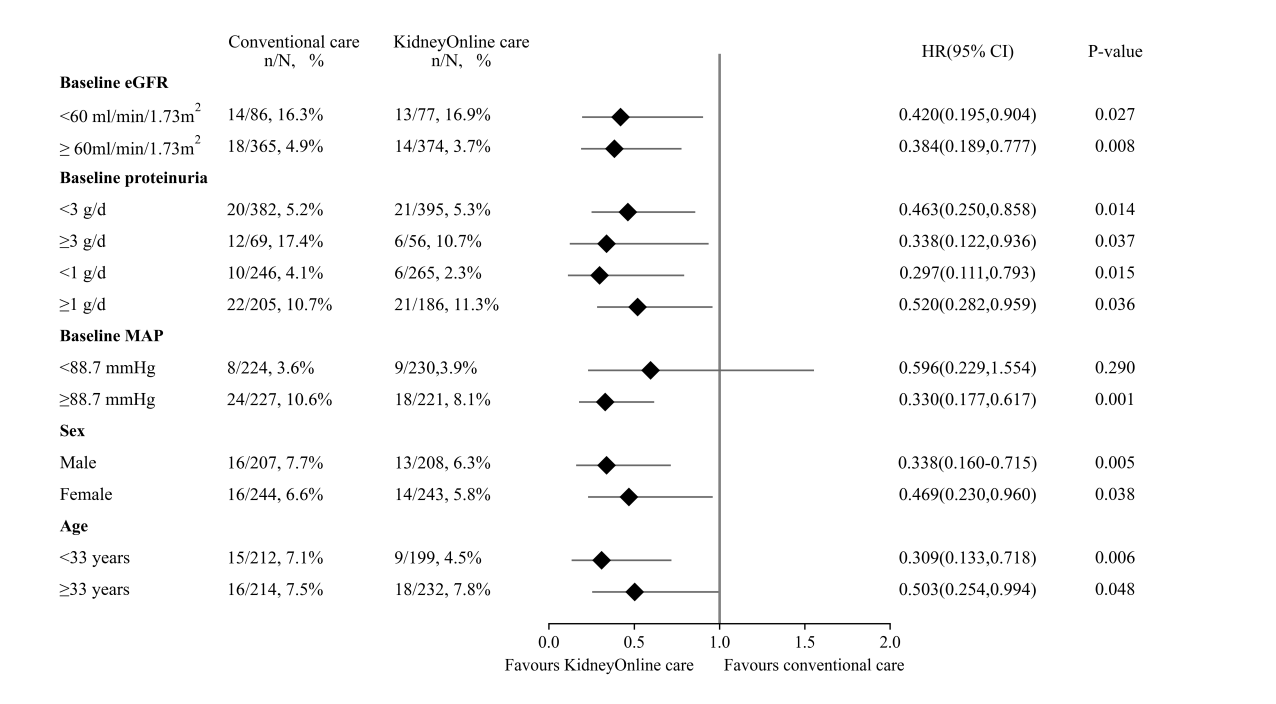

Supplement: Multimedia Appendix 3 [file mhealth_v11i1e45531_app3.png]

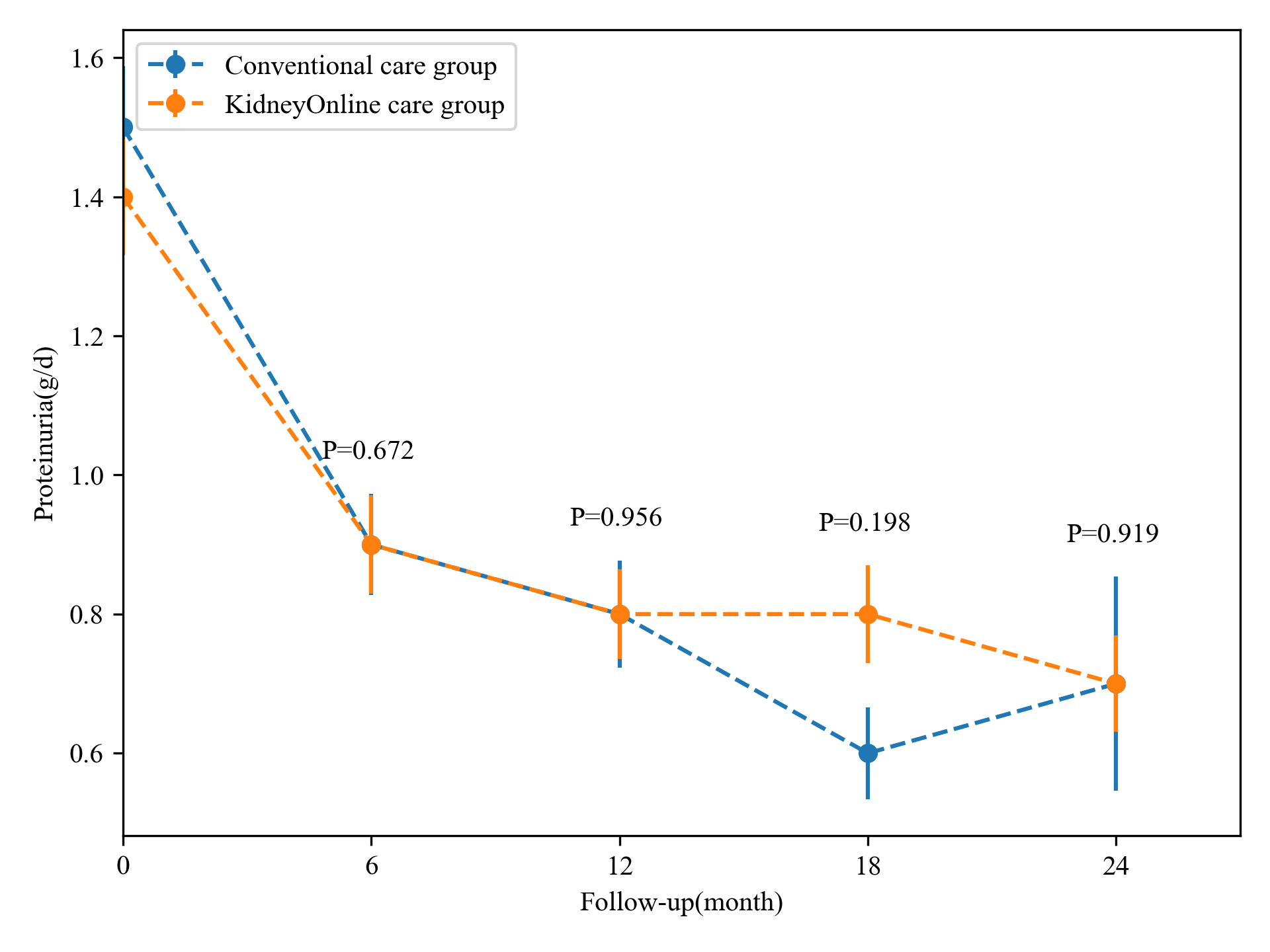

Supplement: Multimedia Appendix 5 [file mhealth_v11i1e45531_app5.png]
