# Supplementary material for: Improving Kidney Outcomes in Patients With Nondiabetic Chronic Kidney Disease Through an Artificial Intelligence–Based Health Coaching Mobile App: Retrospective Cohort Study
Source: JMIR Mhealth Uhealth. 2023 Jun 1;11:e45531. doi: 10.2196/45531 (PMC10273040; doi:10.2196/45531)
Supplement: Multimedia Appendix 4 [file mhealth_v11i1e45531_app4.pptx]

## Slide 1
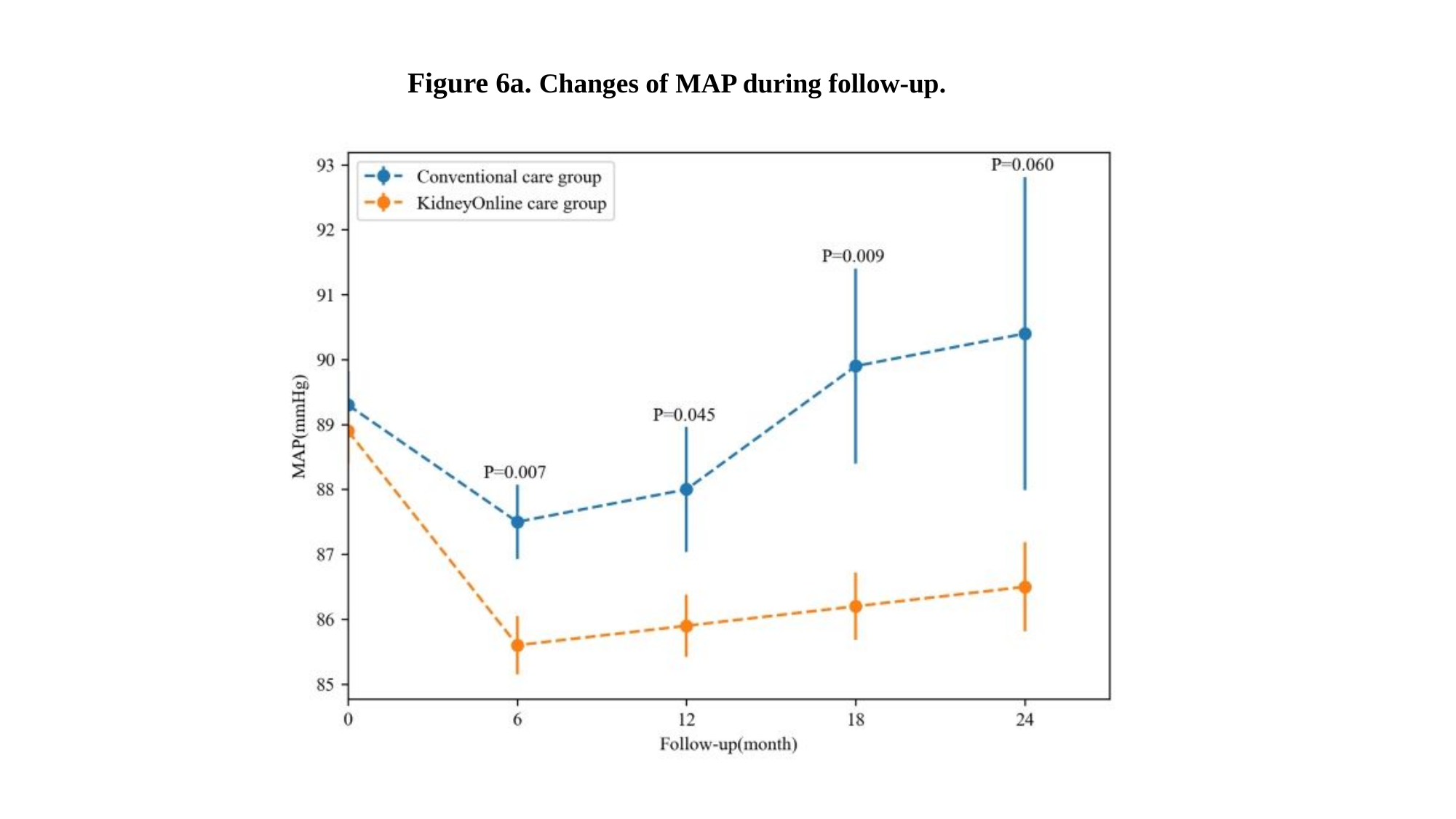

Figure 6a. Changes of MAP during follow-up.

## Slide 2
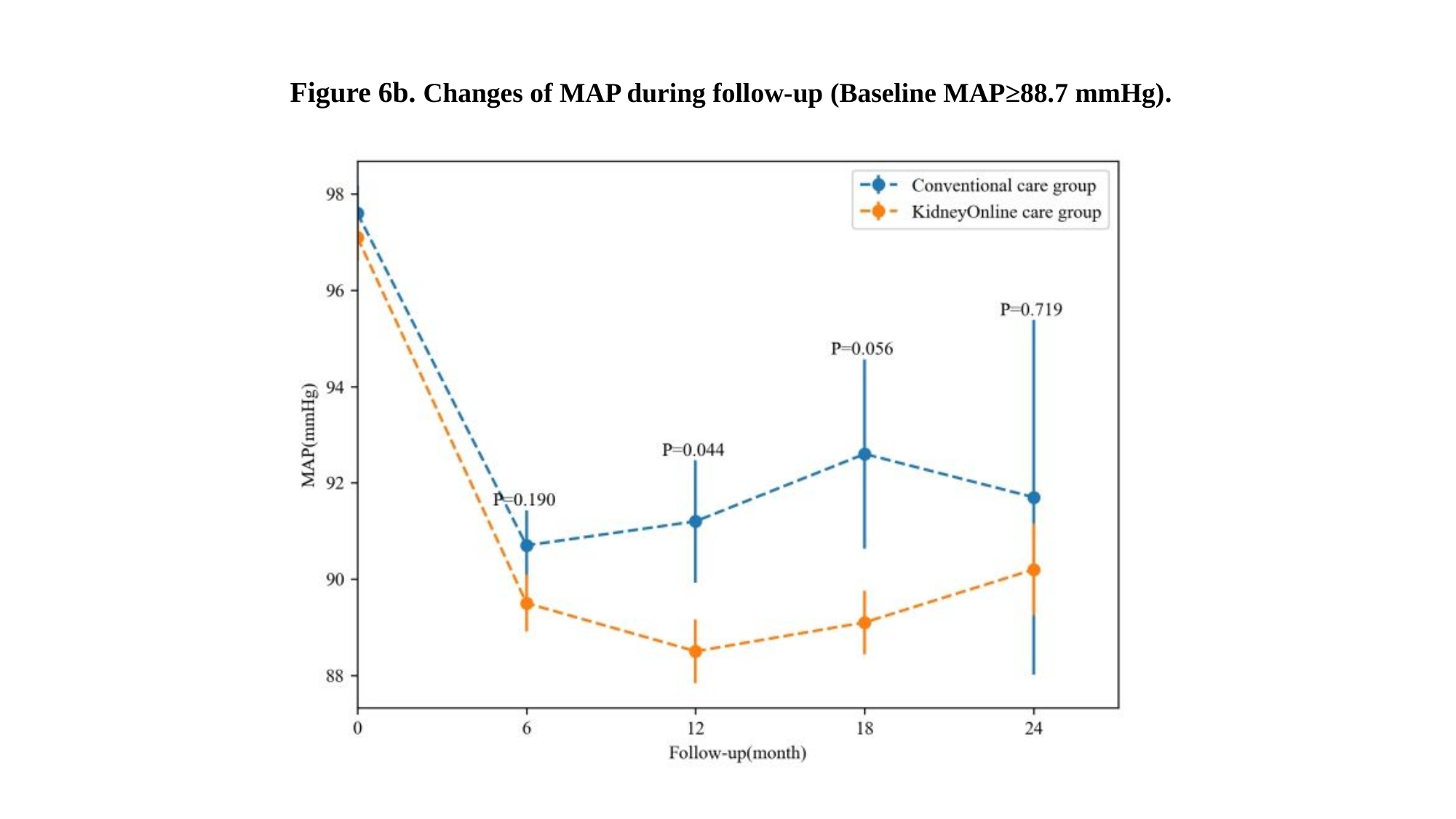

Figure 6b. Changes of MAP during follow-up (Baseline MAP≥88.7 mmHg).

## Slide 3
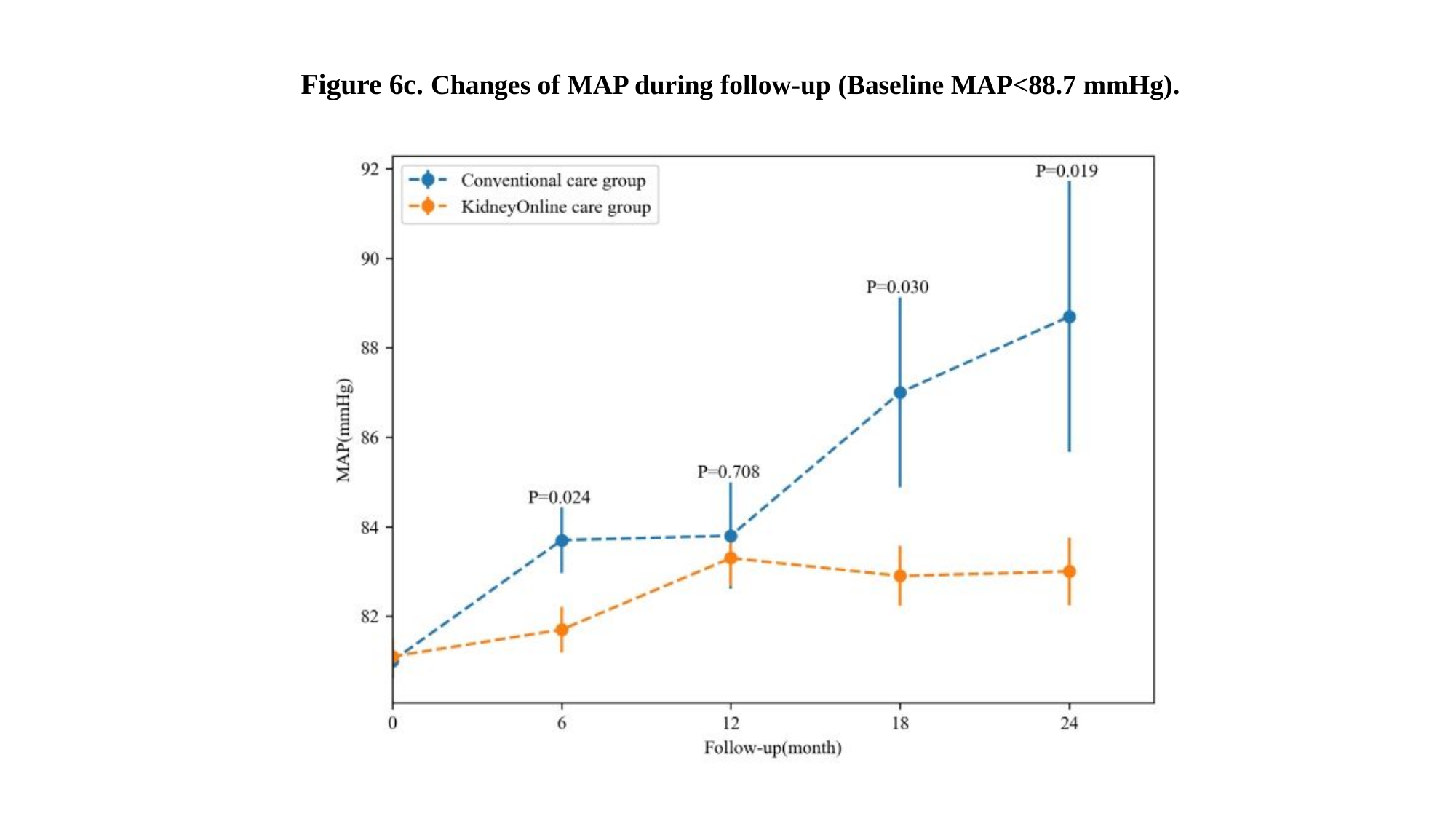

Figure 6c. Changes of MAP during follow-up (Baseline MAP<88.7 mmHg).
